# Skunks as Pets

**Published:** 1885-07

**Authors:** 


### Skunks as Pets.

We have had some encounters with skunks, and seen various dogs debate whether to open an attack upon the "Irishman's Cat," but never made friends with them to any great extent. We have long held to the opinion that the only good skunk was a dead and buried one, and that far away. It is, therefore, with pleasure that I give the experience of my friend, Dr. C. H. Merriam, as related in his Adirondack adventures.

Skunks, particularly when young, make very pretty pets, being attractive in appearance, gentle in disposition, interesting in manners, and cleanly in habits—rare qualities indeed! They are playful, sometimes mischievous, and manifest considerable affection for those who have the care of them I have had, at different times, ten live skunks in confinement. Two summers ago I was the happy master of the cleverest young shunk that I have thus far chanced to meet. For a name, he received the title of his genus, and we called him "Meph," for short. By way of precaution we removed his scent-sacs, and he made a rapid and complete recovery, after a few days of temporary indispoiition. While driving about the country, in the performance of professional duties, he usually slept in my pocket. After supper I commonly took a walk, and he always followed close to my heels. If I chanced to walk to fast for him, he would scold, and stamp his fore-feet; and if I persisted in keeping too far ahead, would turn about, disgusted, and make off in an opposite direction; but if I stopped and called him, he would hurry along at a sort of ambling pace, and soon overtake me. He was particularly fond of ladies, and I think it was the dress that attracted him; but be this as it may, he would invariably leave me to follow any lady that chanced to come near. We used to walk through the woods to a large meadow, which abounded in grasshoppers. Here "Meph" would fairly revel in his favorite food, and it was rich sport to watch his manœuvres. When a grasshopper jumped, he jumped, and I have seen him with as many as three in his mouth and two under his fore-paws at one time. He would eat so many that his over-distended little belly actually dragged upon the ground, and when so full that he could hold no more, would still catch and slay them. When so small that he could scarcely toddle about, he never hesitated to tackle the large and powerful beetle known as the "horned bug," and got many smart nips for his audacity. But he was a courageous little fellow, and it was not long before he learned to handle them with impunity, and it was very amusing to see him kill one. Ere many weeks he ventured to attack a mouse, and the ferocity displayed in its destruction was truly astonishing. He devoured the entire body of his victim, and growled and stamped his feet if any one came near before the repast was over.

His nest was in a box near the foot of the stairs, and before he grew strong enough to climb out by himself, he would, whenever he heard me coming, stand on his hind legs, with his paws resting on the edge of the box, and beg to be carried up stairs. If I passed by without appearing to notice him, he invariably became much enraged, and chippered and scolded away at a great rate, stamping meanwhile most vehemently. He always liked to be carried up to my office, and as soon as strong enough, would climb up of his own accord. He was very sprightly and frolicsome, and used to hop about the floor, and run from room to room in search of something to play with, and frequently amused himself by attempting to demolish my slippers. I have often given him a bit of old sponge, with a string attached, in order to keep him out of mischief. During the evening he occasionally assumed a cunning mood, and would steal softly up to my chair, and, standing erect, would claw at my pants once or twice, and then scamper off as fast as his little legs would carry him, evidently anxious to have me give chase. If I refused to follow, he was soon back, trying a new scheme to attract my attention." Dr. Merriam relates other similar incidents.—*Am. Agriculturist.*

